# Phosphor Deposits of β-Sialon:Eu^2+^ Mixed with SnO_2_ Nanoparticles Fabricated by the Electrophoretic Deposition (EPD) Process

**DOI:** 10.3390/ma7053623

**Published:** 2014-05-06

**Authors:** Chenning Zhang, Tetsuo Uchikoshi, Lihong Liu, Yoshio Sakka, Naoto Hirosaki

**Affiliations:** 1 Materials Processing Unit, National Institute for Materials Science, Tsukuba, Ibaraki 305-0047, Japan; E-Mails: zhang.chenning@nims.go.jp (C.Z.); sakka.yoshio@nims.go.jp (Y.S.); 2 Sialon Unit, National Institute for Materials Science, Tsukuba, Ibaraki 305-0044, Japan; E-Mails: liu.lihong@nims.go.jp (L.L.); hirosaki.naoto@nims.go.jp (N.H.)

**Keywords:** phosphor, light-emitting diodes (LEDs), β-sialon:Eu^2+^

## Abstract

The phosphor deposits of the β-sialon:Eu^2+^ mixed with various amounts (0–1 g) of the SnO_2_ nanoparticles were fabricated by the electrophoretic deposition (EPD) process. The mixed SnO_2_ nanoparticles was observed to cover onto the particle surfaces of the β-sialon:Eu^2+^ as well as fill in the voids among the phosphor particles. The external and internal quantum efficiencies (QEs) of the prepared deposits were found to be dependent on the mixing amount of the SnO_2_: by comparing with the deposit without any mixing (48% internal and 38% external QEs), after mixing the SnO_2_ nanoparticles, the both QEs were improved to 55% internal and 43% external QEs at small mixing amount (0.05 g); whereas, with increasing the mixing amount to 0.1 and 1 g, they were reduced to 36% and 29% for the 0.1 g addition and 15% and 12% l QEs for the 1 g addition. More interestingly, tunable color appearances of the deposits prepared by the EPD process were achieved, from yellow green to blue, by varying the addition amount of the SnO_2_, enabling it as an alternative technique instead of altering the voltage and depositing time for the color appearance controllability.

## 1. Introduction

White light-emitting diodes (LEDs) have attracted much attention due to their low energy consumption, long lifetime, and mercury-free [1]. They have been increasingly used as backlights, automobile headlamps, and architecture lightings by replacing the conventional incandescent and fluorescent lamps [2,3]. The white LEDs mainly consist of a semiconductor chip that emits the blue or near UV (ultraviolet) (nUV) light and photoluminescence (PL) phosphors. These phosphors are desired to absorb the excitation energy from the blue or nUV chip and efficiently yield the visible emissions. Hirosaki *et al.* [4] reported a green phosphor, β-sialon:Eu^2+^, used for the white LEDs. The green-emitting phosphor of the β-sialon:Eu^2+^ is the solid solutions of β-Si_3_N_4_: the host of the β-sialon is formed by the partial replacement of the Si–N with Al–O bonds [5] and the activator of the Eu^2+^ ions are located in the channels along the *c*-direction of the host crystal structure [6]. The β-sialon:Eu^2+^ phosphor can be efficiently excited over a broad wavelength ranging between 280 and 480 nm, which are corresponding to the excitation wavelength by the blue or nUV chips, and has an emission peak at ~540 nm with superior color chromaticity and quantum efficiency [4].

The electrophoretic deposition (EPD) process is based on the movement of the charged particles in the suspension by applying an external electric field. This process has several advantages such as: short depositing time, simple equipment requirement, and little restriction of substrate shape, making it become a cost-effective and versatile process [7,8]. The EPD process has been reported as a widely used technique for preparing a variety of the deposits including coatings [9–11] and ceramic bodies [12–14]. In particular, the EPD process has also been utilized to fabricate the phosphor deposits for the pseudo-white LEDs [15–18], even the monochromatic and color screens for the information displays [19,20]. Moreover, the dependence of the controllability for color appearance for the application in the white LEDs on the deposit thickness could also be achieved by altering the applied voltage and depositing time during the EPD process [15]. However, under the conditions of the high applied voltage and long depositing time, the deposits prepared by the EPD process often have a large sum of voids inside, readily resulting in the peel-off from the substrate due to intense shrinkage after the post thermal treatment.

In this case, an alternative strategy to tune the color appearance of the deposit fabricated by the EPD process becomes necessary. Based on the previous report of the enhancement of the PL intensity and luminescence efficiency by modifying the Ca-α-SiAlON:Eu^2+^ phosphor powders with the SiO_2_ coating [17,18], in this work, another material, SnO_2_, was selected for the coating. The SnO_2_ has colorless presence ascribed to 3.62 eV band gap [21] and refractive index of 2.006 similar to that of ~2.016 of the Si_3_N_4_ (β-sialon). In addition, more interestingly, it has been found that for the application of the SnO_2_, the long wavelength quantum efficiency of a-Si solar cells was greatly enhanced by light scattering at the rough SnO_2_ front contact [22], moreover, when spherical particles were much smaller than the excitation wavelength, all around-spread incident light by Rayleigh scattering may make the irradiation of the phosphor particles become even, therefore leading to the improvement of the excitation efficiency [23,24]. In this study, SnO_2_ spherical nanoparticles (~25 nm) were mixed into the β-sialon:Eu^2+^ suspension used for fabricating the deposits via the EPD process. Through the EPD process depositing, the phosphor deposits of the β-sialon:Eu^2+^ mixed with the SnO_2_ nanoparticles were fabricated. The reported utilization of the Rayleigh scattering of the SnO_2_ nanoparticles would be expected to make the mixed SnO_2_ nanoparticles play a role not only in tuning the color appearance of the phosphor deposit but also improving its luminescence efficiency. Furthermore, for the SnO_2_, the wide band gap resulting in high transparency in the visible light region and similar refractive index to the β-sialon would ensure little influence on the excitation on the phosphor and less emission loss, respectively, In the following sections, the effects of the SnO_2_ addition on the optical properties of the phosphor deposit were investigated.

## 2. Results and Discussion

**Figure 1.**
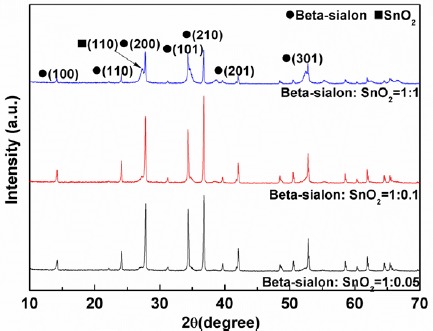
X-ray diffraction (XRD) patterns of the β-sialon:Eu^2+^ deposits, with various mixed amounts (0.05–1 g) of the SnO_2_, prepared by the electrophoretic deposition (EPD) process at 10 s depositing time.

The X-ray diffraction (XRD) patterns of the phosphor deposits, with various mixing amounts (0.05–1 g) of the SnO_2_, prepared by the EPD process at 10 s depositing time are shown in Figure 1. The phase of the β-sialon (JCPDS: 48-1615), the host material of the phosphor, was clearly identified regardless of low mixing amounts (0.05 and 0.1 g) of the SnO_2_ mixture; whereas, the existence of the SnO_2_ diffraction peak with relatively weak intensity was only detected at a high addition amount of 1 g. Due to this reason, it was very difficult to quantify the actual mass ratio of the β-sialon:Eu^2+^ and SnO_2_ in the deposits prepared under various mixing amounts (0.05–1 g) of the SnO_2_.

**Figure 2.**
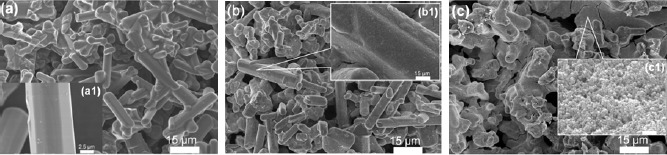
Field-emission scanning electron microscopy (FE-SEM) micrographs of the β-sialon:Eu^2+^ deposits, with various mixed amounts (0.05–1 g) of the SnO_2_, prepared by the EPD process at 10 s depositing time: (**a**) 0.05 g; (**b**) 0.1 g; and (**c**) 1 g; (**a1**), (**b1**) and (**c1**) are their enlarged local images, respectively.

**Figure 3.**
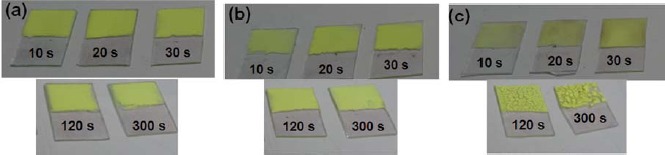
Photographs of the β-sialon:Eu^2+^ deposits, mixed with various amounts (0.05–1 g) of the SnO_2_, prepared by the EPD process at 10–300 s depositing time: (**a**) 0.05 g; (**b**) 0.1 g; and (**c**) 1 g.

Figure 2 demonstrates the field-emission scanning electron microscopy (FE-SEM) images of the phosphor deposits, with various addition amounts (0–1 g) of the SnO_2_, prepared by the EPD process at 10 s depositing time: (a) 0.05 g, (b) 0.1 g, and (c) 1 g. By comparing with the deposit with 0.05 g of the SnO_2_ addition (Figure 2a,a1), it can be obviously found that with 0.1 g addition amount, as shown in Figure 2b, the SnO_2_ nanoparticles were primarily covered onto the particle surfaces of the β-sialon:Eu^2+^ (Figure 2b1). The deposits fabricated with mixing amounts of 0.05 and 0.1 g displayed almost uniform appearances and good adhesion to the subtract under the depositing conditions of 10–300 s, as shown in Figure 3a,b. In contrast, with increasing the addition amount of the SnO_2_ up to 1 g (Figure 2c), most of the mixed SnO_2_ nanoparticles existed inside the voids of the deposited β-sialon:Eu^2+^ particles, as shown in the inset of Figure 2c1. These SnO_2_ nanoparticles were easily shrunk after the thermal treatment, causing the weak adhesion of the deposit to the substrate, especially under the conditions of longer depositing time (120 and 300 s) in this work, as revealed by Figure 3c.

**Figure 4.**
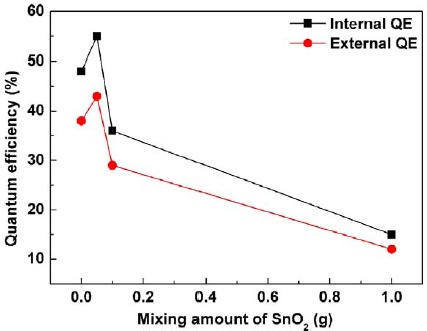
External and internal quantum efficiencies (QEs) of the β-sialon:Eu^2+^ deposits, with various mixed amount of the SnO_2_, prepared by the EPD process at 10 s depositing time as a function of the SnO_2_ addition amount (0–1 g).

Figure 4 presents the external (η_ex_) and internal (η_in_) QEs of the deposits prepared by the EPD process at 10 s depositing time as a function of the mixing amount (0–1 g) of the SnO_2_. The η_ex_ and η_in_ were calculated by using the following equations [25]:

$$\eta_{\text{ex}}=\frac{\int \lambda P(\lambda )d\lambda }{\int \lambda E(\lambda )\text{d}\lambda }$$ (1)

$$\eta_{\text{in}}=\frac{\int \lambda P\left(\lambda \right)d\lambda }{\int \lambda \left[E\left(\lambda \right)-R\left(\lambda \right)\right]d\lambda }$$ (2)

where *E*(λ)/*h*υ, *R*(λ)/*h*υ, and *P*(λ)/*h*υ are the numbers of photons in the excitation, reflectance, and emission spectra of the phosphor, respectively. It was found that the external and internal QEs were both dependent on the addition amount of the SnO_2_. Particularly, by comparing with the deposit without any SnO_2_ mixing (48% internal and 38% external QEs), after mixing the SnO_2_ nanoparticles, with small mixing amount (0.05 g), the both QEs were improved to 55% internal and 43% external QEs. The possible explanation is the utilization of Rayleigh scattering, which is valid in this work due to the size of the used SnO_2_ nanoparticles (~25 nm) much smaller than the excitation wavelength (405 nm), to improve the excitation efficiency for the neighboring phosphor particles upon the SnO_2_ nanoparticles covering on the phosphor particle surfaces [22–24]. However, by increasing the SnO_2_ addition amounts to 0.1 and 1 g, the external and internal QEs were respectively reduced to 36% and 29% for the 0.1 g addition and 15% internal and 12% external QEs for the 1 g addition, which may be ascribed to the diffusive reflection caused by relatively large amount of the SnO_2_ covering the particle surface of the phosphor and therefore resulted in ineffectively excitation on the phosphor [26].

**Figure 5.**
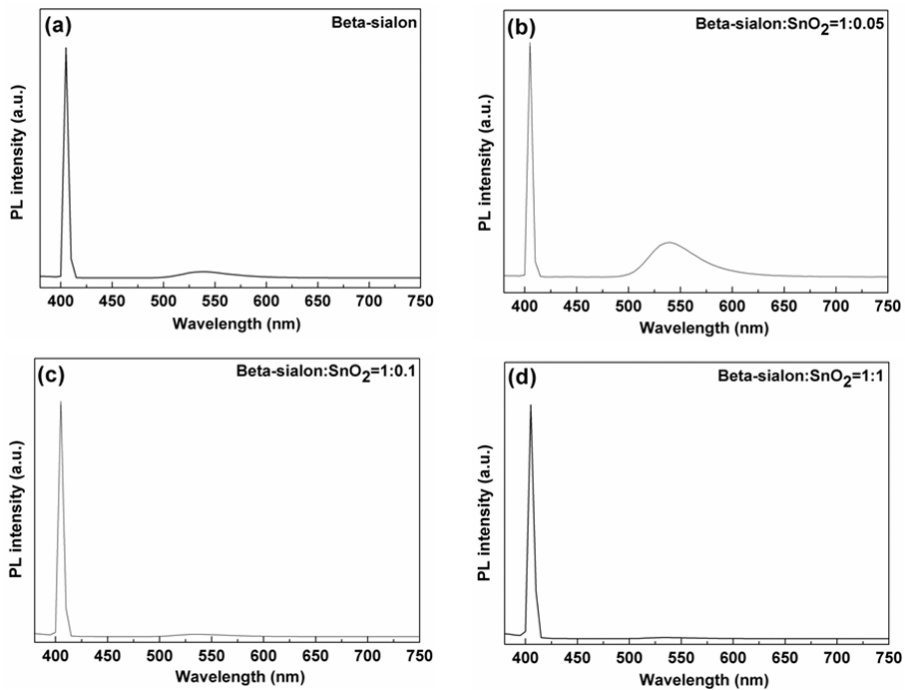
Photoluminescence (PL) spectra of the β-sialon:Eu^2+^ deposits, with various mixed amounts (0.05–1 g) of the SnO_2_ nanoparticles, prepared by the EPD process at 10 s depositing time.

**Figure 6.**
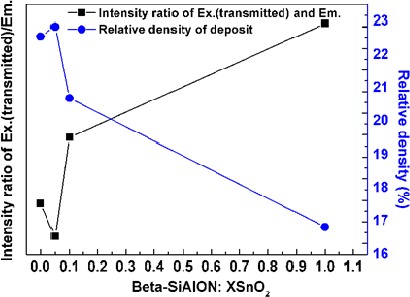
Intensity ratio of the transmitted excitation and emission of the deposit as a function of its relative density by varying the mixing amount of the SnO_2_. The intensities of the transmitted excitation and emission were obtained from the PL spectra (Figure 5).

It has been known that the excitation on the β-sialon:Eu^2+^ is a direct process [27], when the activator of the Eu^2+^ in the host lattice of the β-sialon absorbed enough excitation (405 nm wavelength) energy, the atoms of the Eu^2+^ were lifted up from the ground state (4f^7^) to the excited state (4f^6^5d) by forming valence electrons (e^−^) in the excited state and leaving holes (h^+^) in the ground state. The photons were emitted to yield the PL emission when the excited electrons dropped down to recombine the formed holes (e^−^ + h^+^ → PL emission) in the ground state [26]. From Figure 5, it is noticed that with varying the mixing amount of SnO_2_, the relative intensities of the ~405 nm (transmitted excitation light) and ~540 nm (emission light) peaks varied. The intensity ratio of these two peaks is demonstrated in Figure 6. It is clearly observed that the variation trend of the intensity ratio of the transmitted excitation and emission is inverse-proportionally depending on the relative packing density of the deposit by varying the mixing amount of the SnO_2_, that is, higher relative packing density, lower intensity ratio. In other words, higher relative packing density was in favor of inhibiting the excitation transmitting and therefore of effectively exciting the phosphor to give the emission.

**Figure 7.**
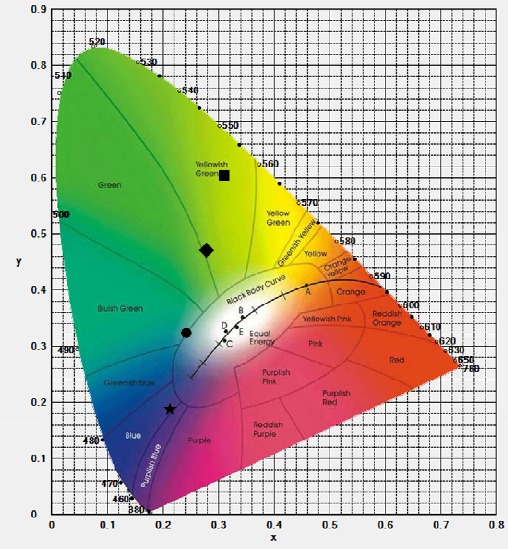
Chromaticity coordinates of the β-sialon:Eu^2+^ deposits, with various mixed amounts (0–1 g) of the SnO_2_, prepared by the EPD process at 10 s depositing time. □, ▪, •, and ★ denote 0, 0.05, 0.1, and 1 g of the SnO_2_ addition, respectively.

By varying the mixing amount (0–1 g) of the SnO_2_, the prepared deposits presented various chromaticity coordinates, which were obtained from their PL intensities of the ~540 nm emissioncombined with the ~405 nm transmitted excitation, of *X* = 0.27, 0.31, 0.24, and 0.21, *Y* = 0.47, 0.61, 0.32, and 0.18 for the deposits with 0, 0.05, 0.1, and 1 g SnO_2_ additions, respectively. The tunable color appearance was achieved from yellow green to blue, as perceived in the chromaticity coordinate (Figure 7). In addition, from Figure 6, it has been known that the deposit having high relative packing density prevented the excitation light from transmitting through the deposit, in turn, made the excitation light effectively excite the phosphor to yield the emission. Since the chromaticity coordinates of the deposits (Figure 7) were obtained based on the intensities of the transmitted excitation and emission (Figure 5), the coordinates of presenting the color appearances of the deposits were also related to their relative packing density, which were controlled by the mixing amount of the SnO_2_, varying from yellowish green to blue. For the color perceive in the chromaticity coordinates diagram, it was rationalized by combining with the lights of the transmitted excitation (~405 nm purplish blue) and emission (~540 nm yellowish green) of the deposits.

**Figure 8.**
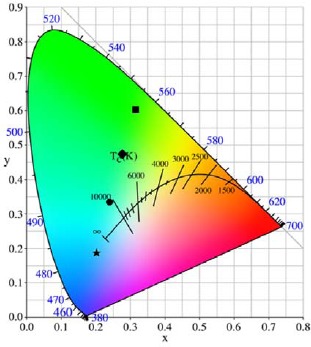
Color temperatures of the β-sialon:Eu^2+^ deposits, with various mixed amounts (0–1 g) of the SnO_2_, prepared by the EPD process at 10 s depositing time. □, ▪, •, and ★ denote 0, 0.05, 0.1, and 1 g of the SnO_2_ addition, respectively.

The color temperatures of the deposits of the β-sialon:Eu^2+^ mixing with various amounts of the SnO_2_ is exhibited in Figure 8. The color temperatures were estimated as 5975 K and 7276 K for the deposits of the β-sialon:Eu^2+^ mixed with 0 and 0.05 g SnO_2_ nanoparticles, respectively, and more than 10000 K for the samples mixed with 0.1 and 1 g SnO_2_. It is obvious that the color temperature is depending on the intensity ratio of the transmitted excitation and emission and therefore dominated by the relative packing density, which can be controlled by varying the mixing amount of the SnO_2_ nanoparticles, of the deposits.

## 3. Experimental Section

**Figure 9.**
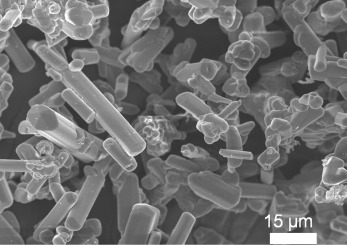
Particle morphology of the as-received β-sialon:Eu^2+^ powders.

The β-sialon:Eu^2+^ phosphor powders, which was received from a Corporation, used in this work have rod-like morphology, with a wide range in length × width of particle distributing from ~2 μm × 10 μm to ~5 μm × 35 μm, as exhibited in Figure 9. One gram of the β-sialon:Eu^2+^ phosphor powder, with mixing various amounts (0–1 g) of the SnO_2_ nanoparticles, which were pre-dispersed in the methanol (50 wt%, Nissan Chemical Industries, Ltd., Tokyo, Japan), were dispersed in 100 mL of the 2-propanol ((CH_3_)_2_CHOH, Nacalai Tesque, Inc., Kyoto, Japan), with adding 2 mL of the distilled water, 2 mL of the glycerol (C_3_H_5_(OH)_3_, Kanto Chemical Co., Inc., Tokyo, Japan), and 0.1 mol of the magnesium nitrate hexahydrate (Mg(NO_3_)_2_·6H_2_O, Kanto Chemical Co., Inc., Tokyo, Japan), under the 30-min ultrasonic stirring.

The prepared suspension was used to fabricate the deposit via the EPD process. For the EPD apparatus, it consists of an ITO (indium tin oxide) glass (10 Ω/cm^2^, model IN-100, Furuuchi Chemical Corporation, Tokyo, Japan) as the cathodic substrate and a stainless steel sheet as the anodic counter electrode. The applied electric field strength between both the electrodes was 50 V/cm and depositing time was ranging from 10 to 300 s. Under the influence of the electric field, the positively charged colloidal particles suspending in the liquid medium were deposited onto the cathodic substrate by the EPD process. The schematic illustration of the depositing in the EPD process has been described in the literature [18]. After the depositing, the deposit was naturally dried in 24 h and then calcined at 425°C for 1 h to convert the formed Mg(OH)_2_ and Mg(C_3_H_7_O)_2_, which are both the products of the reaction: Mg^2+^ + 2(CH_3_)_2_ CHOH = Mg(C_3_H_7_O)_2_ + 2H^+^ and Mg(C_3_H_7_O)_2_+2H_2_O=Mg(OH)_2_+2C_3_H_7_OH, to the MgO as a binder for adhering the deposit to the substrate [19].

**Figure 10.**
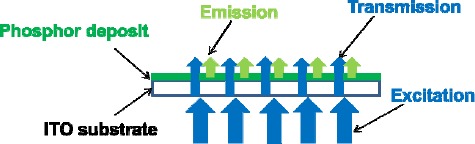
Schematic illustration of the PL measurement. Excitation is incident from one side of the indium tin oxide (ITO) glass without any deposit, and transmitted excitation and emission are collected from the other side of the ITO glass with the deposits.

The phase identification of the powder was performed by X-ray diffraction (XRD) (model RINT 2200, Rigaku Corp., Akishima, Tokyo, Japan) by using nickel-filtered Cu Kα radiation at 40 kV and 40 mA operation with a scanning speed of 0.5°/2θ per minute. The morphology of the deposit was observed by using a field-emission scanning electron microscopy (FE-SEM) (model S-5000, Hitachi, Ltd., Tokyo, Japan). The internal and external quantum QEs of the deposit were conducted by using a multichannel photodetector (model MPCD-7000, Otsuka Electronics, Co., Ltd., Osaka, Japan) with a 200 W Xe-lamp as an excitation source. For the QE measurement, the excitation light from the Xe lamp was filtered by an excitation monochromator (300–600 nm). A white Spectralon standard was illuminated with the resulting monochromatic light. The reflected light was collected by using an integrating sphere and sent to the photodetector for the calibration. The PL property of the deposit was also recorded on the multi-channel photodetector by using 405-nm blue light as the pump source to irradiate from the particle-undeposited side of the substrate. The schematic illustration of the PL measurement is shown in Figure 10.

## 4. Conclusions

The phosphor deposits of the β-sialon:Eu^2+^ mixed with various amounts (0–1 g) of the SnO_2_ nanoparticles were fabricated by the EPD process. By comparing with the deposit without any mixing, after mixing 0.05 g of SnO_2_ nanoparticles, the internal and external QEs of the prepared deposit were both improved; whereas, with increasing the mixing amount to 0.1 and 1 g, they were appreciably reduced. The color appearance tuning from yellow green to blue was achieved by varying the mixing amount of the SnO_2_.
